# School administrator perspectives on electronic nicotine delivery systems use in Montana schools: A mixed-methods study

**DOI:** 10.18332/tpc/224416

**Published:** 2026-07-28

**Authors:** Suzanna Powell, Steven Fuller, Tara Spence, Lisa Richidt, Nicole Aune

**Affiliations:** 1 J.G. Research and EvaluationBozemanUnited States; 2 Montana Department of Public Health and Human ServicesHelenaUnited States

**Keywords:** youth, tobacco prevention, electronic nicotine delivery systems, school administrators, school-based prevention

## Abstract

**Introduction:**

Schools have become central to addressing the prevention, detection, and cessation of youth’s use of Electronic Nicotine Delivery Systems (ENDS). This study surveys and interviews school administrators in Montana to identify effective prevention, discipline, and cessation strategies in schools.

**Methods:**

A mixed-methods study was conducted from March through September 2025, including an online survey with 42 responses from school administrators and 11 follow-up interviews. All Montana school districts (n=400) were invited to participate in the survey, and interview participants were recruited from survey respondents. Survey questions ranged from perceptions of ENDS use to the perception and effectiveness of prevention, discipline, and cessation strategies. Interviews expanded on key survey findings and captured insights into school-level experiences related to ENDS use, prevention, discipline, and cessation strategies.

**Results:**

Of the 400 school districts sent the survey, a response rate of 10.5% (n=42) was achieved. Just over half (61%, n=25) observed student ENDS use on school grounds during the 2024–2025 school year. Certain measures, like implementing student education sessions (90%, n=36) and hallway/bathroom patrols to prevent use and notifying parents for a first offense (95%, n=38) were seen as moderately or very effective. Cessation efforts were more varied, and respondents expressed a desire for more options. The least expensive measures, such as educational sessions, installing tobacco-free signs, and putting up educational posters, were the most commonly implemented measures. In the end, interviews emphasized the need for working collaboratively with peers, parents, and school resource officers to increase prevention, cessation, and disciplinary effectiveness.

**Conclusions:**

Prevention, cessation, and disciplinary strategies to reduce ENDS use at schools vary greatly across Montana. However, respondents consistently expressed a desire for more disciplinary options and cessation resources, and noted that involving peers, parents, and school resource officers in prevention, cessation, and disciplinary strategies increased perceived effectiveness.

## Introduction

Youth use of Electronic Nicotine Delivery Systems (ENDS) has emerged as a persistent public health concern nationally^1^ and in Montana, USA^2,3^. Schools have been tasked with addressing prevention, detection, and response despite rapidly evolving products, shifting regulatory environments, and limited evidence-based guidance tailored to school settings^4^. Although youth ENDS use has been widely studied^5^, less research has examined how schools are responding to ENDS in practice. This study examines how school leaders in Montana address and manage the use of ENDS.

The study had two primary aims: 1) Identify strategies in preventing youth from initiating ENDS use within school settings; and 2) Investigate and understand the implementation of prevention interventions, policies, and procedures at Montana middle and high schools for ENDS.

## Methods

### Study design

This mixed-methods assessment combined quantitative and qualitative approaches to capture a comprehensive understanding of school policies, perceptions, and practices related to student ENDS use across Montana, USA, from the perspective of school administrators. The project was reviewed by the Western IRB (WIRB-Copernicus Group) and found to be exempt from ethical approval.

### Survey

An online survey was sent to principals, superintendents, counselors, and other school administrators in all Montana school districts (n=400) in March 2025. Participation was voluntary, and responses were de-identified prior to analysis. Because the study used a statewide census recruitment approach and was primarily descriptive in nature, no a priori sample size calculation was conducted.

### Quantitative data

Quantitative data were analyzed using RStudio (Version 4.4.1). Descriptive statistics, including frequencies and percentages, were used to summarize school characteristics, prevention measures, disciplinary practices, and perceptions of youth ENDS use. Incomplete responses were excluded, for a final sample of 39 schools. Pair-wise deletion was used to calculate percentages, using the number of respondents to each item as the denominator. Open-ended survey responses were reviewed for recurring themes and used to contextualize quantitative findings.

### Qualitative data

At the end of the survey, respondents were asked to participate in a 45 min follow-up interview. Eleven interviews (four rural and seven urban schools) were conducted with school administrators and staff to expand survey findings and explore school-level experiences, challenges, and promising practices related to e-cigarette use, prevention, and policy enforcement. Interviews were conducted virtually or by phone, recorded with participant consent, and transcribed for analysis. Qualitative data analysis used a hybrid inductive–deductive approach, combining open and closed coding schemas and iterative review to ensure consistency and reliability across interviews.

## Results

Schools included middle school (31%, n=11), high school (36%, n=13), and both (50%, n=18). The number of students enrolled in schools ranged from 4 to 1956 students. Seventeen responses were from rural schools, and 25 were urban. Just over half (61%, n=25) of survey participants reported observing student ENDS use on school grounds during the 2024–2025 school year. Among respondents who observed ENDS use, the most common locations were bathrooms (100%, n=25), parking lots (40%, n=10), and locker rooms (32%, n=8). Most participants viewed vaping as somewhat (51%, n=21) or very problematic (24%, n=10) at their schools. Reducing ENDS use was rated a high priority by 49% (n=20) of participants. Most participants (68%, n=27) reported fewer than one tobacco-related discipline referral per month. Because of the small number of incidents, especially in low-population schools, some do not feel that ENDS use is a priority. However, as one administrator shared:

*‘A lot of kids are using them [e-cigarettes]* … *it’s one of our bigger discipline issues other than probably cell phones.’*

There were 40 responses for what prevention measures are being implemented in schools (Table 1). The most implemented strategies included student education sessions (90%, n=36), hallway/bathroom patrols (83%, n=33), and posting educational materials (83%, n=33).

**Table 1:** Perceived effectiveness of implemented school-based e-cigarette prevention strategies reported by Montana school administrators, March 2025 (N=39)

Prevention measure	Very effective		Moderately effective		Minimally effective		Total
	n	%	n	%	n	%	n
Held educational sessions for students	11	31	16	44	9	25	36
Installed Tobacco-Free School signs	7	19	10	28	19	53	36
Increased hallway or bathroom patrol by teachers	12	36	19	58	2	6	33
Put up educational posters	5	15	13	39	15	46	33
Adopted youth e-cigarette prevention curriculum or program	4	20	7	35	9	45	20
Increased hallway or bathroom patrol by School Resource Officers	7	37	9	47	3	16	19
Held informational meetings for parents/ guardians	5	28	6	33	7	39	18
Installed vape detectors	6	46	5	39	2	15	13

Percentages were calculated within each prevention measure based on the number of respondents who reported implementing that measure (row total n, the total respondents N=39). Totals vary across measures because not all participants reported using every prevention strategy.

For the first offense, the most common consequences were notifying parents (95%, n=38), in-school suspension (63%, n=25), and extracurricular activity restrictions (50%, n=20). For second or third offenses, 87% (n=34) notify parents, but suspensions (in-school: 44%, n=17; out-of-school: 67%, n=26) and law enforcement referrals (54%, n=21) were more common. Many participants (53%, n=21) perceived first offense discipline procedures as ‘moderately effective’ at deterring future ENDS use.

Administrators and School Resource Officers (SROs) expressed frustration over the lack of legal mechanisms when 18-year-old students possess tobacco products at school. Although federal law prohibits tobacco sales to individuals aged < 21 years, state law provides no equivalent possession citation for those aged 18–21 years. Administrators also reported that youth tobacco violations are often perceived as low priority by local authorities. In the words of one administrator:

*‘[local law enforcement] essentially give a free pass for a tobacco possession, which is very unfortunate.’*

Only 25% (n=10) of participants reported that their school used alternative-to-suspension programs for first offenses, though this increased to 41% (n=16) for repeat offenses. Most schools (56%, n=10) using alternative-to-suspension programs considered them ‘moderately effective’ in preventing repeat use. Two schools utilize tobacco education and cessation programs with a local health clinic in their disciplinary response, but this is not accessible to more rural schools.

Regarding vape detector effectiveness, 46% (n=6) of respondents reported they reduced use ‘a little’, 39% (n=5) ‘a lot’, while 8% each (n=1) reported no effect or were unsure. Participants from schools without vape detectors did not show interest in spending money on these devices at this time and consider them cost-prohibitive:

*‘Students have found ways around them. They exhale into a sweatshirt or wet paper towel. And they're pricey. It just seems like a lot of expensive equipment that kids figure out how to bypass quickly.’*

Just under half of the participants reported providing cessation resources to students (46%, n=18), like *My Life, My Quit*. However, many (49%, n=19) participants were unsure how many students wanted to quit vaping. All schools acknowledge that ENDS prevention must be a partnership among students, school employees, parents and family members, community members, law enforcement, behavioral health workers, and others. All schools also leverage positive peer influence by incorporating peer-led education through programs like ReACT, Montana’s youth-led tobacco prevention and advocacy movement, and national initiatives like Drug Prevention Week, as part of a multi-pronged prevention approach.

Participants also expressed concern that students do not understand the true impact of using tobacco products, reporting that students view vapes and other non-combustible forms of tobacco as safer or healthier than smoking cigarettes. As one administrator said:

*‘They all think that the Zyn stuff is safer* … *I think right now they see it as less harmful.’*

School administrators and teachers view this misperception as one of their biggest challenges and report the need for data, resources, and tools to combat this misinformation.

## Discussion

This mixed-methods study provides insight into school-based responses to youth ENDS use in Montana. A survey provided descriptive data on prevention strategies, disciplinary responses, and perceived trends in student vaping behavior, while interviews offered in-depth insights into implementation challenges, effective approaches, and contextual factors influencing school-level practices. Findings suggest that ENDS use continues to present challenges for schools, while prevention efforts, policy enforcement, and resource availability vary across schools. These results align with a growing body of research indicating that although documented tobacco-related disciplinary incidents may appear relatively low, student ENDS use remains visible within school environments^6^. ENDS use was most frequently observed in semi-private spaces such as bathrooms, reinforcing concerns that traditional monitoring and disciplinary approaches may not capture the scope of use^7^. Consistent with prior literature, administrators perceived student vaping as problematic and reported mixed confidence in the effectiveness of existing prevention and disciplinary strategies^8^.

These results echo broader evidence suggesting that punitive responses alone are imperfect deterrents, particularly when misperceptions about the safety of ENDS products persist^9^. Administrator interest in alternatives-to-suspension parallels emerging policy and research advocating for educational and restorative approaches over exclusionary discipline^8^. Taken together, this study underscores that youth ENDS use is not solely a behavioral issue but also a structural and informational challenge.

### Limitations

Several limitations should be considered when interpreting these findings. Voluntary participation may have led to selection bias by disproportionately capturing the perspectives of schools more engaged in tobacco prevention efforts. The survey instrument was informed by previous literature but not formally validated, possibly contributing to measurement bias through differences in question interpretation. Additionally, survey and interview responses were self-reported and therefore subject to social desirability and recall biases. The low response rate (10.5%) introduces a potential non-response bias. Participating schools may differ systematically from non-participating schools in policies, resources, or perceptions of youth ENDS use.

### Implications

Additional work is needed to evaluate the effectiveness of school-based prevention and cessation strategies, particularly alternatives to suspension and peer- or education-based approaches. Future research should evaluate the long-term effectiveness of school-based prevention policies and collaborative prevention efforts involving schools, families, and community stakeholders, while also assessing strategies targeting emerging nicotine products and how evolving products influence youth behaviors and prevention outcomes.

## Conclusions

This study offers practical insight into how schools navigate student ENDS use within operational and policy constraints. Findings are broadly consistent with prior research highlighting the difficulty of prevention, detection, and sustained behavior change^4,10,11^. Results underscore the need for flexible, comprehensive approaches to address changing patterns of youth tobacco use.
